# *In Vitro* and *In Vivo* Activity of an Organic Tellurium Compound on *Leishmania (Leishmania) chagasi*

**DOI:** 10.1371/journal.pone.0048780

**Published:** 2012-11-07

**Authors:** Isabella Aparecida Salerno Pimentel, Carolina de Siqueira Paladi, Simone Katz, Wagner Alves de Souza Júdice, Rodrigo L. O. R. Cunha, Clara Lúcia Barbiéri

**Affiliations:** 1 Departamento de Microbiologia, Imunologia e Parasitologia, Escola Paulista de Medicina - Universidade Federal de São Paulo, São Paulo, São Paulo, Brazil; 2 Centro Interdisciplinar de Investigação Bioquímica, Universidade de Mogi das Cruzes, Mogi das Cruzes, São Paulo, Brazil; 3 Centro de Ciências Naturais e Humanas, Universidade Federal do ABC, Santo André, São Paulo, Brazil; Technion-Israel Institute of Technology, Israel

## Abstract

Tellurium compounds have shown several biological properties and recently the leishmanicidal effect of one organotellurane was demonstrated. These findings led us to test the effect of the organotellurium compound RF07 on *Leishmania (Leishmania) chagasi*, the agent of visceral leishmaniasis in Latin America. *In vitro* assays were performed in *L. (L.) chagasi*-infected bone marrow derived macrophages treated with different concentrations of RF07. In *in vivo* experiments Golden hamsters were infected with *L. (L.) chagasi* and injected intraperitoneally with RF07 whereas control animals received either Glucantime or PBS. The effect of RF07 on cathepsin B activity of *L. (L.) chagasi* amastigotes was assayed spectrofluorometrically using fluorogenic substrates. The main findings were: 1) RF07 showed significant leishmanicidal activity against intracellular parasites at submicromolar concentrations (IC50 of 529.7±26.5 nM), and the drug displayed 10-fold less toxicity to macrophages (CC50 of 5,426±272.8 nM); 2) kinetics assays showed an increasing leishmanicidal action of RF07 at longer periods of treatment; 3) one month after intraperitoneal injection of RF07 *L. (L.) chagasi*-infected hamsters showed a reduction of 99.6% of parasite burden when compared to controls that received PBS; 4) RF07 inhibited the cathepsin B activity of *L. (L.) chagasi* amastigotes. The present results demonstrated that the tellurium compound RF07 is able to destroy *L. (L.) chagasi in vitro* and *in vivo* at concentrations that are non toxic to the host. We believe these findings support further study of the potential of RF07 as a possible alternative for the chemotherapy of visceral leishmaniasis.

## Introduction

Leishmaniasis comprise a group of diseases whose etiological agents are protozoan parasites of the *Leishmania* genus that cause cutaneous, mucocutaneous and visceral leishmaniasis. According to the World Health Organization there are currently 12 million cases of leishmaniasis and 350 million people are at risk of acquiring some form of the disease. *Leishmania (Leishmania) chagasi* is the causative agent of visceral leishmaniasis (VL) in Latin America. VL is the most severe form of the disease and affects 500,000 people worldwide. VL is a chronic, debilitating disease which has a consumptive character, and may lead to death if untreated [1], [2].

**Figure 1:**
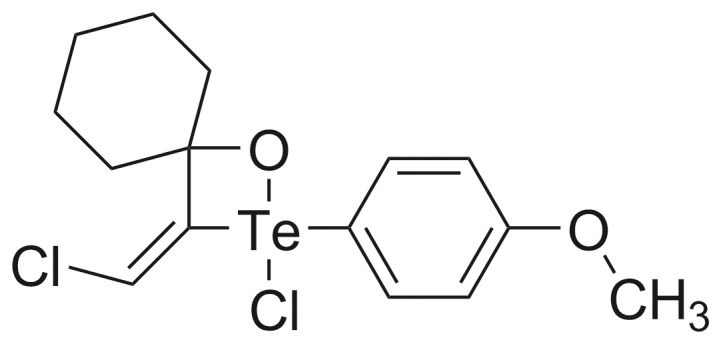
Molecular structure of organotellurane RF07.

The first-line drugs used for treatment of leishmaniasis are pentavalent antimonial compounds, while amphotericin B and pentamidine are used as the second-line chemotherapy [3]. Miltefosine, originally developed as an anticancer agent, showed good efficacy for treatment of visceral leishmaniasis in India and cutaneous leishmaniasis in Colombia [4], [5]. However, the use of these compounds is limited by toxicity to the host and the development of resistance by the parasites [3], [6]. Furthermore, dogs, the main reservoir of zoonotic VL in Latin America and Mediterranean area, do not respond satisfactorily to drugs used for human VL chemotherapy. For this reason the control of canine visceral leishmaniasis has been based on the sacrifice of infected animals that results in a serious problem for public health agents and an ineffective reservoir control that contributes to disease urbanization [7]. These issues have led to the search of new drugs less toxic to VL patients and also active against canine VL. Several compounds including synthetic, natural products extracted from plants and marine sources have shown different degrees of efficacy in the treatment of experimental VL [8]–[12].

During the last decade an increasing interest in the study of tellurium compounds as chemotherapeutic agents has emerged. There are two main classes of tellurium compounds which comprise inorganic and organic derivatives that have shown antimicrobial, antihelmintic, antioxidant, immunomodulatory and antitumoral properties [13]–[18]. Hypervalent organotellurium compounds termed organotelluranes have also been studied as potent inhibitors of cysteine proteases [14], [19] and their effect on systems associated to the activity of these enzymes has been demonstrated. Thereby, the effect of organotelluranes in angiogenesis, in the cytotoxicity on several cancer cell lineages, in the induction of apoptosis in human HL-60 cells, as well as in the inactivation of caspases leading to the protective effect of epilepsy in rats was reported [20], [21]. In *Leishmania* cathepsin B has been found to be involved in parasite invasion and survival in macrophages [22], [23]. The inhibitory role of organotelluranes on cathepsin B activity and recent demonstration that the organotellurane RT01 destroyed promastigote and amastigote forms of *L. (L.) amazonensis* [24] led us to test the leishmanicidal activity of the organotellurane RF07 on *L. (L.) chagasi*. The present study describes the effect of this tellurium compound on intracellular amastigotes and Golden hamsters infected with *L. (L.) chagasi.*

## Methods

### Animals

Female BALB/c mice six to eight weeks old were obtained from breeding stocks maintained at Universidade Federal de São Paulo (São Paulo, Brazil) and eight-week-old male Golden hamsters were acquired from Universidade de Campinas (São Paulo, Brazil). This study was carried out in strict accordance with the recommendations in the Guide for the Care and Use of Laboratory Animals of the Brazilian National Council of Animal Experimentation (http://www.cobea.org.br). The protocol was approved by the Committee on the Ethics of Animal Experiments of the Institutional Animal Care and Use Committee at the Federal University of São Paulo (Id # CEP 1295/10).

**Figure 2:**
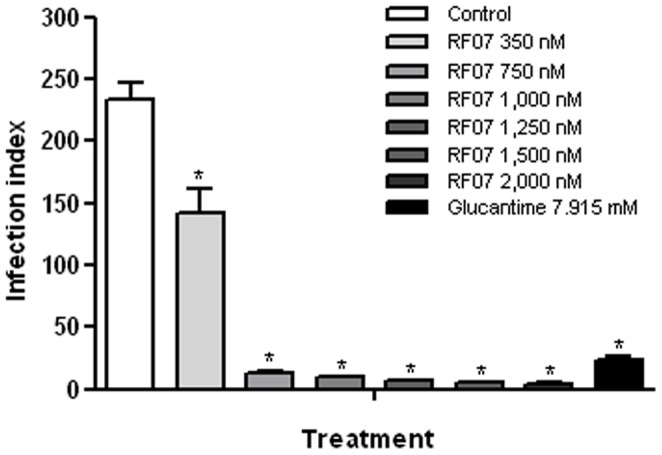
Activity of RF07 on *L. (L.) chagasi*-infected macrophages. Mouse bone marrow derived macrophages were infected with amastigotes of *L. (L.) chagasi*, treated with the drugs for 3 days and the infection index was estimated. ^*^ *P*<0.001 compared to control.

### Parasites

The *L. (L.) chagasi* strain used (MHOM/BR/1972/LD) was kindly provided by Dr. Jeffrey J. Shaw, Instituto Evandro Chagas (Belém, Pará, Brazil) and maintained as amastigotes by inoculation of Golden hamsters by the intraperitoneal route every 4 to 6 weeks as previously described [25]. Two months after infection, the animals were sacrificed and the spleens were homogenized and centrifuged for isolation of amastigotes. The resulting pellet was resuspended in phosphate buffered saline (PBS), centrifuged at 250×*g* for 5 min, the supernatant was centrifuged at 1,400×*g* for 5 minutes and the pellet was resuspended in PBS. The suspension was agitated for 3 hours at room temperature and centrifuged at 1,400×*g* for 5 minutes. The final pellet contained purified amastigotes essentially free of contamination by other cells and macrophage debris. This pellet was resuspended in PBS, amastigotes were counted in a hemocytometer and used to infect macrophages or hamsters.

### Hypervalent Tellurium Compound 4-{2-Chloro-3-[chloromethylidene]-1-oxa-2-λ^4^-telluraspiro[3.5]non-2-yl}phenyl Methyl Ether (RF07)

The tellurium compound RF07 (molecular weight 427.5 g/mol) (Figure 1) was synthesized by reaction of *p*-methoxyphenyl tellurium with 1-ethynyl-1-cyclohexanol in benzene reflux as previously described [26]. After preparation, the product RF07 was firstly purified by fractional crystallization and then re-purified by re-crystallization for use in biological assays. Stock solutions prepared in dimethylsulfoxide (DMSO) were diluted with PBS for use in the *in vitro* and *in vivo* assays.

**Figure 3:**
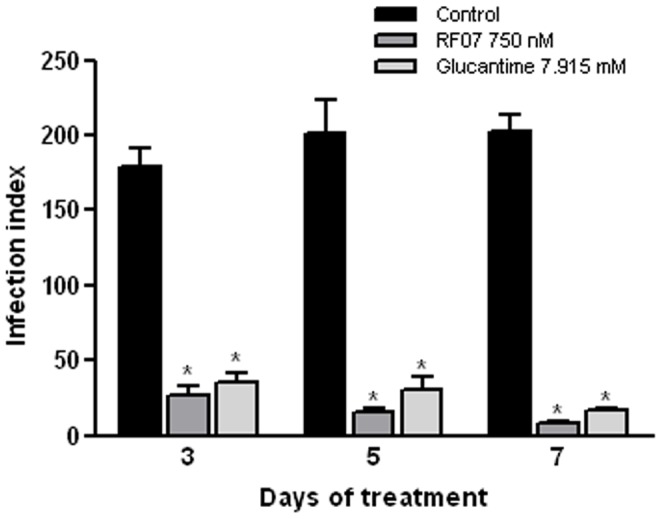
Kinetics of RF07 leishmanicidal activity on *L. (L.) chagasi*-infected macrophages. Macrophages were infected with amastigotes of *L. (L.) chagasi*, treated with the drugs for 3, 5 and 7 days and the infection index was calculated after each period. ^*^ *P*<0.001 compared to control.

### Effect of RF07 on *L. (L.) chagasi*-infected Macrophages

The activity of RF07 on intracellular amastigotes was evaluated in mouse bone marrow derived macrophages infected with *L. (L.) chagasi*. Bone marrow derived macrophages were generated from bone marrow stem cells isolated from BALB/c mice [27]. Cells were counted, added (8×10^5^) and cultured on glass coverslips inserted in 24-well tissue culture plates containing RPMI 1640 medium buffered with 15 mM of HEPES, 20 mM of sodium bicarbonate and supplemented with 1 mM of L-glutamine, 20% of fetal calf serum (FCS) and 30% of L929 cell conditioned medium (LCCM). Cultures were kept at 37°C in an atmosphere of air/CO_2_ (95/5%). After 5 days, the medium was changed for RPMI containing 10% of FCS and macrophages were infected at a multiplicity of 5 amastigotes per macrophage. After 24 hours, infected cultures were treated with different drug concentrations (350 to 2,000 nM) for 3 days. The coverslips were fixed with methanol, stained with hematoxylin-eosin (HE) and intracellular amastigotes were counted. Results are expressed by the infection index, obtained by multiplying the percentage of infected macrophages by the average number of amastigotes per macrophage. At least 200 macrophages were scored in each 3 coverslips. For kinetics experiments *L. (L.) chagasi*-infected macrophages were treated with 750 nM of RF07 for 3, 5 and 7 days and the infection index was evaluated. Glucantime (Sanofi-Aventis, Brazil, 300 mg/ml, 81 mg/ml Sb^V^) was used as standard drug for treatment of *L. (L.) chagasi* amastigotes.

### Cytotoxicity Assays

RF07 cytotoxicity to macrophages was tested by a MTT micromethod described previously [28] after incubation of bone marrow derived macrophages with 350 nM to 10 µM of RF07 for 3 days. Macrophages were also incubated with the highest concentration of DMSO used for RF07 solubilization (0.04%). The formation of formazan was measured by adding 3-(4,5-dimethylthiazol-2-yl)-2,5-diphenyltetrazolium bromide (MTT; Molecular Probes, Eugene, OR, USA) 0.5 mg/ml and incubation of the cultures at 37°C in the dark. After 4 hours the medium was removed, 200 µl of DMSO was added per well and the absorbance was measured using an ELISA reader at 540 nm (Labsystems Multiskan).

### Antileishmanial *in vivo* Activity

In order to evaluate the *in vivo* leishmanicidal activity of RF07 male Golden hamsters 8 weeks-old were infected by intraperitoneal route with 1×10^8^ *L. (L.) chagasi* amastigotes freshly isolated from spleen of infected hamsters. One month after infection, the animals were randomly separated in 5 groups of 6 hamsters each. Treated animals received daily intraperitoneal doses of either 52.76 µmol/kg/day of Glucantime or 0.323 µmol/kg/day, 0.646 µmol/kg/day or 1.292 µmol/kg/day of RF07 for 15 days. Control group received the same number of injections of PBS. One month after treatment the animals were sacrificed and the parasite burden was evaluated in spleen and liver by limiting dilution method, as previously described [29].

### Toxicity Assays for Golden Hamsters

Serum concentrations of albumin, transaminases and creatinine were determined in Golden hamsters 15 days after the end of treatment, using sets of commercial reagents (Doles Reagentes e Equipamentos para Laboratórios, Ltda, Brazil).

**Figure 4:**
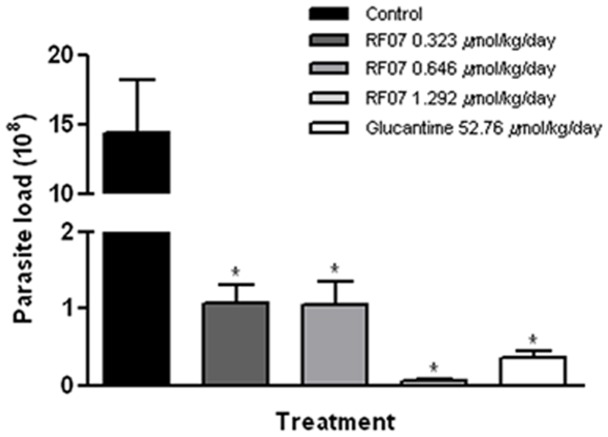
Treatment of *L. (L.) chagasi*-infected hamsters with RF07. Spleen parasite load of *L. (L.) chagasi*-infected hamsters treated with either different concentrations of RF07 or Glucantime was determined by the limiting dilution method one month after end of the treatment. ^*^ *P*<0.001 compared to control.

### Assays of Cathepsin Activity in *L. (L.) chagasi* Amastigote Lysates

Cathepsin activities were monitored with the fluorogenic substrates Z-Phe-Arg-AMC (for all cathepsins) and Z-Arg-Arg-AMC (for cathepsin B) using 10 µl of *L. (L.) chagasi* amastigote cell lysate (1×10^9^ amastigotes disrupted in 100 µl PBS), 1 ml of four-component buffer comprised of 25 mM acetic acid, 25 mM MES (4-morpholineethanesulfonic acid), 75 mM Tris, 25 mM glycine, pH 5.0, 5 mM dithiothreitol (DTT), 10 µM of each fluorogenic substrate and 50 µM of RF07. The effect of RF07 on the parasite enzyme activity was tested by incubation of the *L. (L.) chagasi* lysate with RF07 for 2 minutes in buffer solution at 37°C; the fluorogenic substrate was then added and fluorescence of the released fluorophore, 7-amino-4-methylcoumarine (AMC), was measured over time. The remaining enzyme activities were determined and expressed as a percentage of the activity of the control experiment. Parasite lysate was also incubated with 6,4 µM of the fluorogenic substrate Abz-Gly-Ile-Val-Arg-Ala-Lys(Dnp)-OH (Sigma, St. Louis, MO, USA), specific for cathepsin B [30], in the presence of either increasing concentrations of RF07 or CA074, a specific inhibitor of cathepsin B. The cathepsin activity was monitored spectrofluorometrically using the fluorogenic substrates on a Hitachi F-2000 spectrofluorometer equipped with a thermostated cell holder. The fluorescence excitation (λEx) and emission (λEm) wavelengths, for the fluorescence of AMC, were set at 380 nm and 460 nm, respectively, while the parameters for the fluorescence of Abz-peptide fragments resulting from the Abz-Gly-Ile-Val-Arg-Ala-Lys(Dnp)-OH hydrolysis were set at λ_ex_ = 320 and λ_em_ = 420 nm.

**Figure 5:**
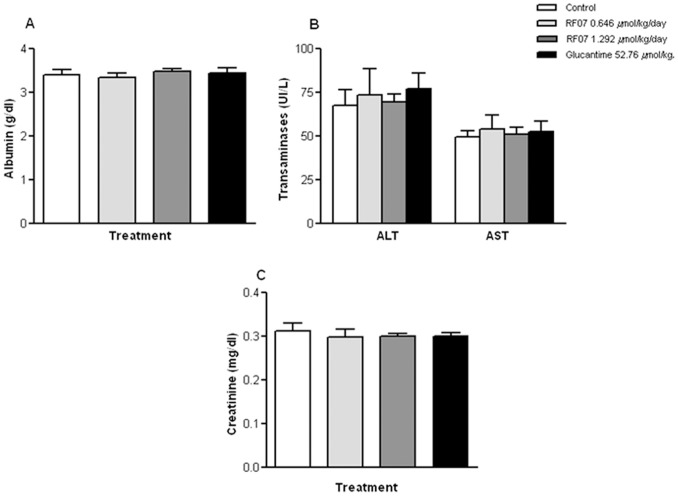
Toxicity evaluation in *L. (L.) chagasi*-infected hamster after treatment with RF07. Serum concentrations of albumin (A), transaminases (B) and creatinine (C) in *L. (L.) chagasi*-infected hamsters 15 days after treatment with either RF07 or Glucantime. The reference values are: albumin: 3.2–4.3 g/dl; transaminases: ALT: 53–202 UI/L, AST: 28–107 UI/L; creatinine: 0.5–0.6 mg/dl.

### Statistical Analysis

ANOVA and Student’s *t* test were used to determine the statistical differences between groups and *P* values <0.05 or lower were considered statistically significant. IC50, IC90, CC50 and CC90 values were determined by GraphPad Prism, version 5.0.

**Figure 6:**
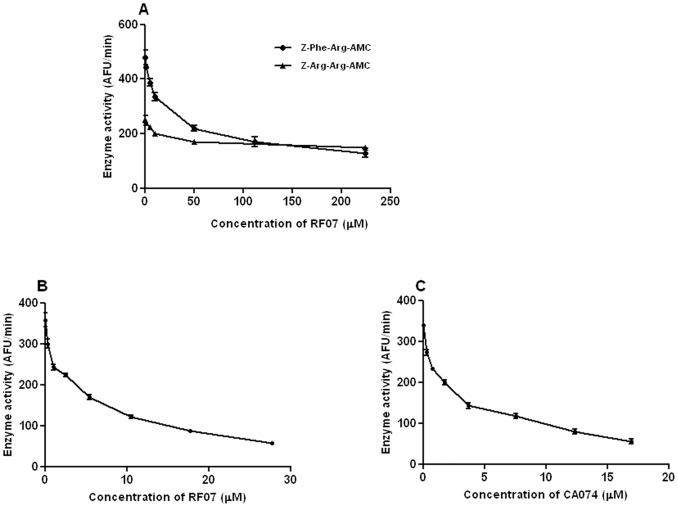
Effect of RF07 on proteolytic activity of *L. (L.) chagasi* amastigotes. A - Fluorogenic substrates with different specificities for cathepsin-like proteases were incubated with extracts of *L. (L.) chagasi* amastigotes in the presence of DTT and increasing concentrations of RF07, as indicated. B and C - Fluorogenic substrate specific for cathepsin B-like proteases was incubated with extracts of *L. (L.) chagasi* in presence of increasing concentrations of either RF07 (B) or CA074 (C).

## Results

### Leishmanicidal Activity of RF07 on *L. (L.) chagasi*-infected Macrophages

Mouse bone marrow derived macrophages infected with *L. (L.) chagasi* amastigotes for 24 hours were treated with different concentrations of RF07 ranging from 350 to 2,000 nM. Treatment of infected cultures with Glucantime was used as a positive control. Three days after incubation, infected macrophages were fixed and stained for parasite counts and determination of the infection index. A significant, dose-dependent decrease in macrophage infection was observed with an inhibition of 85% for 750 nM of RF07 (IC50 of 529.7±26.5 nM and IC90 of 1,748±335 nM) (Figure 2). The cytotoxicity of RF07 on macrophages was evaluated by the MTT method and 96 h after treatment a CC50 of 5,399±290 nM and a CC90 of 17,905±1.050 nM were determined. It is still important to mention, as expected from the chemical stability of the hypervalent tellurium compounds [14], [15], that 3 different batches of RF07 were tested and no differences in their leishmanicidal effect were observed (data not shown). Leishmanicidal effect on intracellular amastigotes was also observed in the presence of Glucantime tested in a range of 15–600 µg/ml and IC50 and IC90 values were 12.62 and 113.5 µg/ml, respectively (corresponding to 33±4.1 µM and 296±35 µM).

Kinetics of *L. (L.) chagasi* destruction was performed by use of RF07 at 750 nM for 3, 5 and 7 days. An inhibition of 85%, 92.4% and 95.7% on *L. (L.) chagasi* infection was observed 3, 5 and 7 days after treatment, respectively (Figure 3). Glucantime also exhibited a significant leishmanicidal effect (80%, 84.9% and 91.5%), but was toxic to macrophages after 5 and 7 days, while RF07 did not show macrophage toxicity in any period of treatment (data not shown).

### Effect of RF07 on Hamsters Infected with *L. (L.) chagasi*

Golden hamsters infected with *L. (L.) chagasi* and treated with either 0.323 µmol/kg/day, 0.646 µmol/kg/day or 1.292 µmol/kg/day of RF07 displayed a significant reduction of spleen parasite load of 92.5%, 92.6% and 99.6%, respectively (Figure 4). Control groups that received 52.76 µmol/kg/day of Glucantime showed a reduction of 97.5% compared to untreated controls. Similar results were observed in liver parasite burden.

Evaluation of hepato and nephrotoxicity of RF07 by detection of serum levels of albumin, transaminases and creatinine showed no statistically significant alterations between non-treated and treated groups (Figure 5).

### Effect of RF07 on Proteolytic Activity of *L. (L.) chagasi* Amastigotes

In order to investigate the possible inhibitory action of RF07 on *L. (L.) chagasi* cysteine proteases, spectrofluorometric assays were performed by incubation of cathepsin-like cysteine protease substrates with extracts of *L. (L.) chagasi* amastigotes in the presence of the reducing agent DTT. Although a dose-dependent inhibition of *L. (L.) chagasi* proteolytic activity by RF07 was observed by using both fluorogenic substrates (Figure 6A), a value of IC50 ten times higher was obtained on Z-Arg-Arg-AMC hydrolysis (IC50 of 450 µM and 41 µM for Z-Arg-Arg-AMC and Z-Phe-Arg-AMC, respectively). However, at lower concentrations RF07 inhibited the activity of *L. (L.) chagasi* extract on a most specific substrate for cathepsin B-like (Figure 6B). Inhibition of *L. (L.) chagasi* proteolytic activity by CA074 was also observed (Figure 6C) and the calculated IC50 values for RF07 and CA074 were not significantly different (4.47±0.13 µM and 2.41±0.15 µM, respectively). These results strongly suggest that RF07 inhibits *L. (L.) chagasi* cathepsin B-like activity.

## Discussion

The present study focused on the *in vitro* and *in vivo* activity of the organotellurane RF07 on *L. (L.) chagasi*. Our data showed the leishmanicidal activity of RF07 against intracellular amastigotes, whereas the drug displayed 10-fold less toxicity to macrophages. Although similar leishmanicidal effect was observed with Glucantime, significantly higher concentrations of the antimonial were necessary to destroy *L. (L.) chagasi* amastigotes. The leishmanicidal activity of RF07 is comparable to that reported for several compounds tested against visceralizing *Leishmania* species like the 2,4,6-trisubstitued pyrimidines and 1,3,5-triazines, tamoxifen and several drugs tested by high-throughput screening such as naloxonazine and others not identified [31]–[34]. However, higher concentrations of these compounds were used to destroy *L. (L.) chagasi*, whereas RF07 exerted an effective leishmanicidal effect at submicromolar concentrations. Among other tellurium compounds previously tested against *Leishmania* only the organotellurane RT01 exhibited an *in vitro* and *in vivo* antileishmanial property [24]. Neverthless, this compound was tested on a cutaneous species of *Leishmania*, impairing the comparison with RF07 data.

The efficiency of RF07 in destroying *L. (L.) chagasi in vivo* was also demonstrated. Treatment of *L. (L.) chagasi*-infected hamsters with RF07 led to a significant reduction of parasite load in animal spleens (92.5%, 92.6%, and 97.5%, respectively, with 0.323 µmol/kg/day, 0.646 µmol/kg/day, and 1.292 µmol/kg/day of RF07). Although the reduction of parasite burden in *L. (L.) chagasi*-infected hamsters treated with RF07 was similar to that observed with Glucantime, the antimonial compound was used in 40 times higher concentration. These data support literature reports that show the high concentrations of Glucantime required for a curative effect in patients suffering from visceral leishmaniasis that result in the toxicity often observed among these patients [35]. On the other hand, treatment with RF07 did not result in toxicity in *L. (L.) chagasi*-infected hamsters as demonstrated by hepatic and renal assays after treatment with the drug according to reported reference values [36]. The leishmanicidal effect of RF07 on *L. (L.) chagasi*-infected hamsters is also comparable to that obtained with tamoxifen, whereas it showed higher effectiveness than 2,4,6-trisubstitued pyrimidines and 1,3,5-triazines that reduced the parasite burden by 48 to 52%. Moreover, in both assays infected hamsters received significantly higher drug concentrations than those used for treatment with RF07 [33], [34].

At this stage, our data pointed out the use of RF07 as a leishmanicidal agent. However, it is important to consider the possible occurrence of cross-resistance of RF07 and Sb-resistant strains. This hypothesis is based on the mechanisms of action and resistance of antimonial-based compounds toward *Leishmania* which involves the reactions of the Sb(III) species with biothiols [38], similar to the known particular reactivity of telluranes towards thiols [19], [37], [39].

Literature evidence that organotelluranes inhibit cysteine protease activity [19], [37] led us to test the effect of RF07 on *L. (L.) chagasi* protease activity. Our previous spectrofluorometric assays indicated an apparent inhibition of parasite cathepsin L. However, cloning of the gene encoding cathepsin B of *L. (L.) chagasi* and analysis of predicted amino acid sequence of this enzyme showed a replacement of serine 331 by a glycine residue which results in a low activity of this enzyme toward the synthetic peptide substrate Z-Arg-Arg-AMC and favours the hydrolysis of the substrate Z-Phe-Arg-AMC [22]. These data led us to test a more specific substrate for cathepsin B whose hydrolysis was significantly inhibited in presence of low concentrations of RF07. The involvement of *L. (L.) chagasi* cathepsin B with mouse macrophage infection and parasite survival has been already demonstrated [22], [23]. Thus, our findings may indicate a relationship between the leishmanicidal effect of RF07 and *L. (L.) chagasi* cathepsin B inhibition. However, other relevant targets may account for the leishmanicidal effect of the drug and they are now under investigation. Tellurium compounds have exhibited immunomodulatory properties [15]. Studies on the possible immunomodulatory effect of RF07 in treated *L. (L.) chagasi*-infected hamsters are currently in progress.

In conclusion, the effectiveness of RF07 in destroying *L. (L.) chagasi in vitro* and *in vivo* at concentrations non toxic to the host opens perspectives to explore the potential of RF07 as an additional option for the chemotherapy of visceral leishmaniasis, encouraging us to extend these studies for treatment of canine visceral leishmaniasis.
